# The Impact of Timing of Concurrent Chemoradiation in Patients With High-Grade Glioma in the Era of the Stupp Protocol

**DOI:** 10.3389/fonc.2019.00186

**Published:** 2019-03-27

**Authors:** Kwanza T. Warren, Linxi Liu, Yang Liu, Michael T. Milano, Kevin A. Walter

**Affiliations:** ^1^School of Medicine and Dentistry, University of Rochester Medical Center, Rochester, NY, United States; ^2^Department of Public Health Sciences, University of Rochester Medical Center, Rochester, NY, United States; ^3^Department of Neurosurgery, University of Rochester Medical Center, Rochester, NY, United States; ^4^Department of Radiation Oncology, University of Rochester Medical Center, Rochester, NY, United States

**Keywords:** gliobastoma, high-grade glioma, chemoradiation, timing, wait time

## Abstract

**Background:** The purpose of this study is to provide a critical review of current evidence for the impact of time to initiation of chemoradiation on overall survival in patients with newly diagnosed high-grade gliomas treated with radiation and concurrent temozolomide chemotherapy.

**Methods:** A literature search was conducted using PubMed/MEDLINE and EMBASE databases. Studies were included if they provided separate analysis for patients treated with current standard of care: radiation and concurrent temozolomide. Bias assessment was performed for each included study using the Newcastle-Ottawa Assessment Scale, with Karnofsky Performance Status (KPS) and extent of resection used for comparability.

**Results:** The initial search yielded 575 citations. Based on the inclusion/exclusion criteria, a total of 10 retrospective cohort studies were included in this review for a total of 30,298 patients. Of these, one study described an indirect relationship between time to initiation of treatment and overall survival. One study found decreased survival only with patients with significantly longer time to treatment. Four studies found no significant effect of time to treatment on overall survival. The four remaining studies found that patients with moderate time to initiation had the best overall survival.

**Conclusion:** This review provides evidence that moderate time to initiation of chemoradiotherapy in patients with high-grade gliomas does not lead to a significant decrease in overall survival, though the effect of significant delays in treatment initiation remains unclear.

## Introduction

Glioblastoma (GBM) is the most common primary central nervous system tumor in adults, accounting for 45.2% of malignant primary brain tumors in the United States (1). The current standard of care that provides the greatest life expectancy in these patients became standard of care following the publication by Stupp et al. and includes maximal safe tumor resection followed by radiation therapy with concurrent temozolomide (TMZ) for 6 weeks and six subsequent cycles of adjuvant TMZ (2). In the European Organisation for Research and Treatment of Cancer (EORTC) and National Cancer Institute of Canada (NCIC) randomized study, this regimen (referred to as the Stupp protocol) was associated with an increase in median survival of GBM patients from 12.1 to 14.6 months when compared to the previous standard of radiotherapy alone (3). In this trial, patients had a median time from diagnosis to start of chemoradiotherapy of 5 weeks (range: 1.7–12.9 weeks), however the optimal timing of initiation of chemoradiation has not been well elucidated.

Studies of optimal timing of radiation therapy in breast, lung, and head and neck cancers have consistently shown an indirect correlation between time to initiation of radiation and recurrence risk (4–7). For aggressive malignancies such as GBM with rapid doubling time, it would be expected that longer time to initiation of treatment could allow for further tumor growth and progression (8). Indeed, studies have shown areas of increased contrast enhancement between the time of tumor resection and the time of therapy initiation consistent with tumor progression in 82% of patients (9).

Due to ethical concerns, no prospective trials have been conducted to address the question of optimal timing of treatment initiation in patients with GBM. Several retrospective studies that have attempted to address this question have yielded conflicting results (10). Some studies have found that increasing time from surgical resection to initiation of treatment is correlated with worse overall survival (11–13), while other studies have found no association between the timing of treatment and patient outcomes (14–16). Some studies have even shown a potential benefit to moderately increased time to treatment initiation, though a mechanism for this phenomenon has not been well established (17, 18). Many of the aforementioned studies took place prior to the initiation of the Stupp protocol in 2005 and all systematic reviews and meta-analyses on the topic include studies that were done prior to this time period.

The purpose of this study is to provide a critical review of the current evidence for the impact of time to treatment (TT) initiation of chemoradiation on overall survival of patients with GBM who were treated with the current standard of concurrent radiation and TMZ.

## Methods

This systematic review adheres to the Preferred Reporting Items for Systematic Reviews and Meta-Analyses (PRISMA) protocol (19).

### Search Strategy

The goal of this search was to identify all published works evaluating the effect of timing of initiation of post-operative chemoradiotherapy in patients with high-grade gliomas (grade III/IV) treated with the current standard of care. The databases used for this search included the U.S. National Library of Medicine (PubMed/MEDLINE) and Excerpta Medica Database (EMBASE). All searches were limited to January 2005–June 2018, as the EORTC/NCIC randomized study was published in 2005 (2). Key words used in the search algorithm included: glioma, glioblastoma, radiotherapy, chemoradiotherapy, timing, early, and delay. Specific search algorithms were designed in accordance with the author and an institutional research librarian (Table S1). All citations of the articles selected in the initial screening of search results were manually evaluated for eligibility as well.

### Selection Criteria

Eligibility criteria included publications that evaluated overall survival as it related to time between surgical resection and initiation of chemoradiotherapy in adult patients with newly diagnosed high-grade gliomas (grade III/IV). Publications were eligible if they included patients who underwent adjuvant treatments other than adjuvant chemoradiation with TMZ, as long as patients who underwent combined radiation therapy with temozolomide therapy were analyzed separately. Publications were excluded if they included patients with recurrent gliomas, patients who did not undergo a neurosurgical procedure (either biopsy, subtotal or gross total resection), or patients who did not undergo combined radiation and temozolomide therapy within the analysis.

Titles and abstracts were reviewed from the initial search and excluded publications that were clearly inappropriate. After duplications were removed, all remaining publications underwent full-text inspection to evaluate eligibility based on the aforementioned criteria.

### Data Collection

The following information was collected from each study: study period, total sample size, patient ages, Karnofsky performance status (KPS), tumor histology, extent of resection, and adjuvant chemotherapy and radiation regimens. Each of the included publications divided patients into different subgroups based on the time between surgery and initiation of therapy. Hazard Ratios with 95% confidence interval were collected from each study. Any additional factors that were found to be significantly associated with overall survival were recorded as well. For those studies that included it, information regarding factors associated with early and/or delayed treatment initiation was also recorded.

### Risk of Bias Assessment

Bias assessment for individual studies was evaluated using the Newcastle-Ottawa Quality Assessment Scale for Cohort studies, which evaluates studies based on selection (maximum of 4), comparability (maximum of 2), and outcome (maximum of 3) (20, 21). Factors included in the “comparability” category included control for KPS and extent of resection, as each of these factors has consistently been shown in multiple studies to be highly associated with prognosis (22–24).

## Results

**Figure 1:**
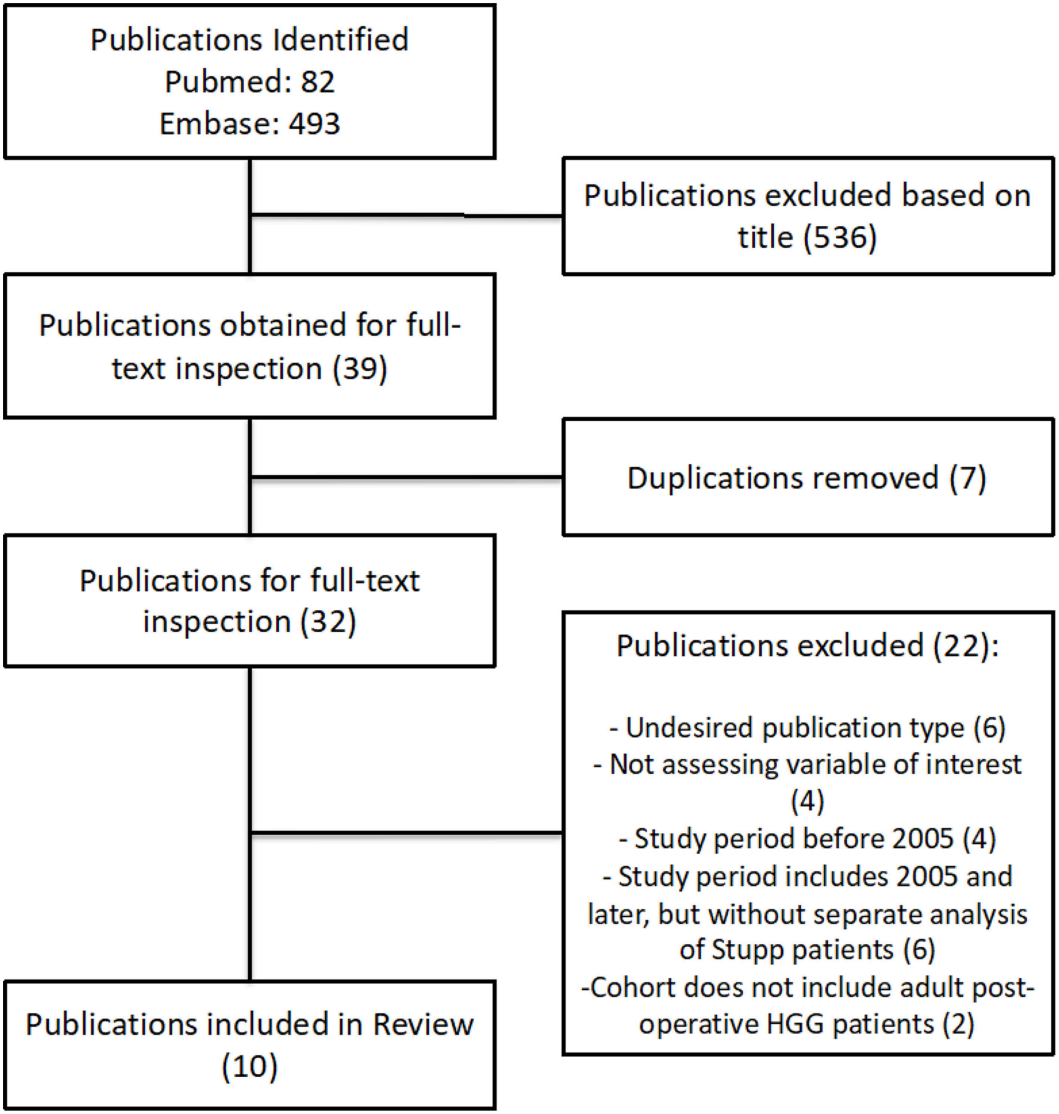
Literature search strategy, results, and selection criteria. The search was performed on July 3, 2018. HGG, high-grade gliomas.

A total of 575 citations resulted from the initial database search of which 32 were selected for full-text inspection following exclusion based on title/abstract and removal of duplications. Of these 32 publications, 10 met inclusion criteria and were included in this systematic review. Reasons for exclusion include publication type, not assessing variable of interest, study period prior to 2005, study period includes patients treated after 2005 but without separate analysis of patients receiving the Stupp protocol, and cohort that does not include post-operative high-grade glioma (HGG) patients (Figure 1). With these 10 retrospective cohort studies combined, a total of 30,298 patients were assessed. Three of the studies evaluated patients who had been enrolled in various clinical trials for correlation between TT and overall survival (OS) (25–27). Nine of the studies were done exclusively in patients with grade IV gliomas, and one study cohort was a mix of grade III and grade IV gliomas with the majority of patients being grade IV. Information regarding the study period, total number of patients, patient ages, KPS scores, tumor histology, extent of resection, chemotherapy regimen, radiation dosages, median TT and TT subgroups for each study can be found in Table 1.

**Table 1:** Study characteristics of all studies included in this systematic review.

	**Study period**	**Patients (n)**	**Patient ages (median [range])**	**KPS (median)**	**Histology (% GBM)**	**Extent of resection**	**Chemo regimen (% receiving TMZ)**	**Radiation**	**Median TT (days)**	**TT subgroups**
Adeberg et al. (26)	2004–2011	177	58.8 [20.3–75.9]	90	100	• Biopsy: 4% • STR: 60% • GTR: 36%	86.4%	60 Gy	31^*^	< 24 d > 24 d
Blumenthal et al. (25)	2011	1,395	58 ^*^ [19–87]	90	100	• STR: 42.9% • GTR: 53.0% • Other: 4.1%	100	60 Gy	26^*^	≤ 3 wks 3–4 wks > 4 wks
Han et al. (27)	2004–2010	198	55.1 [21.3–80]	90	100	• Biopsy: 16.7% • STR: 47.9% • GTR: 33.8%	100	60 Gy	29.5	< 30 d 30–34 d > 34 d
Louvel et al. (28)	2005–2011	692	Mean: 57.5 ± 10.8	34.2% ≤ 70 65.8% >70	100	• Biopsy: 0% • STR: 34.5% • GTR: 65.5%	100	60 Gy	42	< 1.5 mos > 1.5 mos
Nathan et al. (29)	2005–2014	2,535	58^*^	Not reported	77	Not reported	100	60 Gy	35.7^*^	0–4 wks 4–6 wks 6–13 wks
Noel et al. (15)	2006	400	60.5 [22.7–85.6]	Not reported	100	• Biopsy: 36% • STR: 23% • GTR: 41%	100	60 Gy [median]	41	2–4 wks 5 wks 6 wks 7 wks ≥ 8 wks
Osborn et al. (30)	2010–2012	11,652	61 [IQR: 53–69]	Not reported	100	• Biopsy: 0% • STR: 55.1% • GTR: 44.9%	100^**^	Not reported	30	≤ 24 d 25–30d 31–37d > 37d
Pollom et al. (31)	2010–2013	12,738	61–69	Not reported	100	• Biopsy: 22% • STR: 37% • GTR: 41%	100^**^	27% < 60 Gy 66% ≥ 60 Gy	29	< 15d 15–21d 22–28 d 29–35 d 36–42 d > 42 d
Sun et al. (32)	2005–2015	218	58 [21–86]	80	100	Not reported	100	60 Gy	27	< 27 > 27
Wang et al. (33)	1996–2014	447	23.5% < 50 76.5% ≥ 50	80.3% ≥ 70 19.7% > 70	100	• Biopsy: 21.5% • STR: 14.5% • GTR: 64%	61%	10.3% < 36 Gy 9.8% 36–54 Gy 79.9% > 54 Gy	34% < 21 33.7% 21–32 32.2% > 32	< 21 d 21–32 d > 32 d

* Median reported as average of medians for groups involved, as data regarding the entire cohort was unavailable

** *Type of chemotherapy not recorded, but assumed to be TMZ by authors given time period*.

*D, days; wks, weeks; mos, months; KPS, karnofsky performance Score; GBM, glioblastoma multiforme; TMZ, temozolomide; WT, wait time; STR, subtotal resection; GTR, gross total resection*.

**Figure 2:**
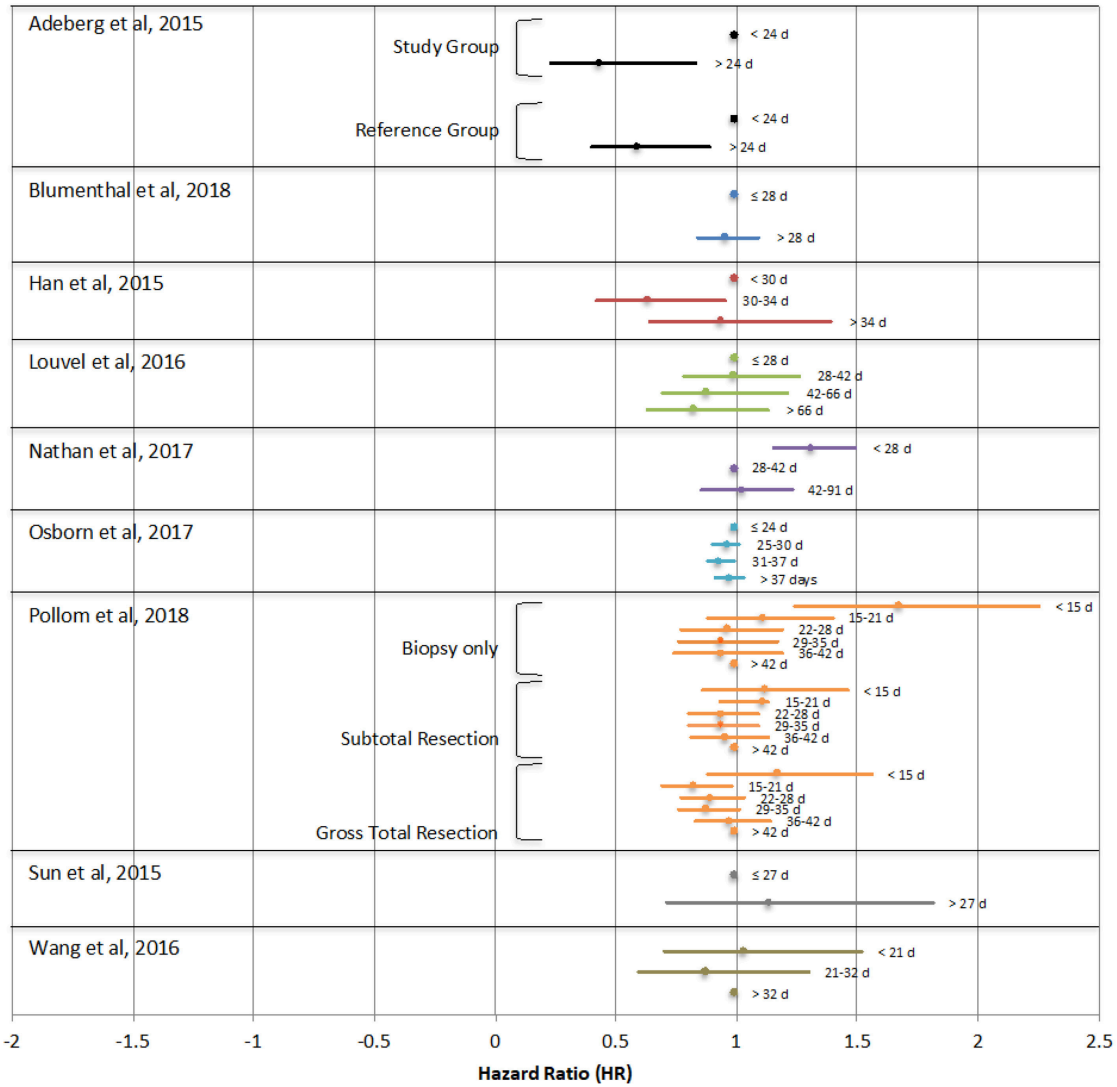
Hazard ratios (HR) of overall survival as they relate to time between surgical resection and initiation of chemoradiotherapy reported in each study. The point depicted with HR = 1 was used as the reference group. Any HR > 1 denotes an increased risk of death. Noel et al. is not indicated in this figure as this study did not report hazard ratios as they relate to treatment time.

Of the studies analyzed, one study found improved survival with early initiation of treatment (within 15–21 days) compared to longer time to initiation (>42 days) only in patients who underwent gross total resection, though the opposite was true for patient who underwent biopsy only (31). One study found poorer survival only in a small subset of patients with particularly long TT (>6 weeks) (32). Four studies found no statistically significant effects of TT on OS (15, 25, 28, 30). Adeberg et al, Han et al, Nathan et al, and Wang et al. each found that the greatest survival was in patients with a slight delay to chemoradiotherapy initiation of >24 days, 30–34 days, 4–13 weeks, or 21–32 days respectively (26, 27, 29, 33). Figure 2 demonstrates the hazard ratio of death of study groups reported in each study relative to their respective reference points, which are indicated by HR of 1. Noel et al is not indicated in this figure, as it did not report HR as it relates to TT. This study found no statistically significant differences in median survival in patients with TT of 2–4 weeks, 5 weeks, 6 weeks, 7 weeks or ≥8 weeks (15).

Other variables found to be significantly associated with survival included O6-methylguanine-DNA methyltransferase (MGMT) promoter methylation status in 3 studies (25, 26, 30), recursive partitioning analysis (RPA) classification in 3 studies (25, 28, 33), sex in 4 studies (25, 28–30), age in 4 studies (27, 29, 30, 32), KPS in 2 studies (27, 33), and extent of resection in 4 studies (27, 28, 30, 33). Additionally, Osborn et al found significant associations between survival and Charlson/Deyo comorbidity score, non-white race, tumor size, and facility type (academic vs. non-academic) (30). Wang et al also found significant associations between overall survival and total RT dose and use of TMZ (33). The hazard ratios for each of these associations are detailed in Table 2.

**Table 2:** This table depicts the values that were found to be significantly related to overall survival in each study that reported these variables as well as their corresponding hazard ratios and 95% confidence intervals.

	**Factors associated with OS on multivariate analysis**	**Hazard ratio (95% CI)**	***P*-value**
Adeberg et al. (26)	MGMT promoter methylation	0.43 (0.18,0.99)	0.048
Blumenthal et al. (25)	RPA IV (vs. III)	1.65 (1.37, 1.99)	<0.001
	RPA V (vs. III)	2.91 (2.34, 3.61)	<0.001
	MGMT unmethylated	1.72 (1.48, 2.00)	<0.001
	Male sex	1.31 (1.14, 1.50)	<0.001
Han et al. (27)	Age	1.03 (1.02,1.05)	<0.001
	KPS	3.64 (1.55,8.55)	0.003
	Biopsy (vs. STR/GTR)	2.93 (1.93, 4.45)	<0.001
Louvel et al. (28)	Male sex	1.28 (1.06,1.55)	0.012
	RTOG-RPA class 5–6	1.31 (1.08,1.58)	0.005
	Total resection (vs. partial)	0.75 (0.62,0.91)	0.004
Nathan et al. (29)	Age at craniotomy	1.031 (1.026,1.036)	<0.0001
	Female sex	0.837 (0.742,0.944)	0.0038
Osborn et al. (30)	Age > 60	1.68 (1.61,1.75)	<0.001
	Charlson/Deyo 1 (vs. 0)	1.17 (1.10,1.24)	<0.001
	Charlson/Deyo ≥ 2 (vs. 0)	1.37 (1.27,1.47)	<0.001
	Female gender	0.90 (0.87,0.94)	<0.001
	Other race (vs. white)	0.68 (0.60,0.78)	<0.001
	Tumor size 3–5cm (vs. < 3)	1.09 (1.03,1.16)	<0.001
	Tumor size >5cm (vs. < 3)	1.13 (1.06,1.20)	<0.001
	MGMT methylation	0.72 (0.65,0.81)	<0.001
	GTR (vs. STR)	0.82 (0.79,0.86)	<0.001
	Academic facility	0.91 (0.87,0.95)	<0.001
Sun et al. (32)	Age	1.018 (1.001,1.036)	0.049
Wang et al. (33)	KPS < 70	3.586 (1.644,7.822)	0.001
	Biopsy only (vs. GTR)	2.510 (1.327,4.747)	0.005
	RPA class IV (vs. III)	3.467 (1.351,8.898)	0.01
	RPA class V/VI (vs. III)	3.650 (1.077,12.369)	0.001
	Total RT dose < 36 Gy (vs. >54)	4.671 (2.241,9.737)	0.001
	No temozolomide	3.823 (1.694,8.627)	0.001

*MGMT, O6-methylguanine-DNA methyltransferase; RPA, recursive partitioning analysis; KPS, Karnofsky performance status; STR, subtotal resection; GTR, gross total resection; RT, radiation therapy*.

Five of the included studies analyzed factors that are significantly associated with early and/or delayed treatment, which are outlined in Table 3. In Han et al. it was found that patients with biopsy only were significantly more likely to start treatment earlier and younger patients were more likely to start treatment later. Louvel et al. reported that patients were more likely to have longer TT if they had a carmustine wafer implantation during surgery. Patients in this cohort with shorter TT were more likely to be RPA class 5 or 6, have neurologic deficits, or have post-operative epileptic seizures. In Osborn et al. patients with shorter TT were more likely to be treated at non-academic facilities, be of white race, have lager tumors, and have subtotal resection (vs. GTR). In Wang et al. patients with shorter TT were more likely to be older, have lower KPS, have biopsy only, have a higher RPA class or have a 3-dimensional conformal RT or 2-dimensional RT technique. Pollom et al. (31) found associations with longer TT in patients who were black/African-American, had Medicaid/government insurance/no insurance, lived in a metropolitan area, or lived >50 miles from the treatment facility. Patients in this cohort were more likely to have a shorter TT if they had a higher income.

**Table 3:** This table depicts all of the variables that were found to be statistically significantly associated with longer or shorter TT in the five studies that reported this analysis.

	**Association**	**Variable**	**P value**
Han et al. (27)	Shorter TT	Biopsy only	0.006
	Longer TT	Younger age	0.02
Louvel et al. (28)	Shorter TT	Carmustine wafer implantation	<0.001
	Longer TT	RPA class 5–6 Neurologic deficit Post-operative seizures	<0.001 <0.001 0.049
Osborn et al. (30)	Shorter TT	Non-academic treatment facility White race Larger tumor size STR (vs. GTR)	0.002 <0.001 <0.001 <0.001
Wang et al. (33)	Shorter TT	Older age Lower KPS Biopsy only Higher RPA class RT technique 3D conformal or 2D	0.006 <0.001 <0.001 <0.001 0.007
Pollom et al. (31)	Shorter TT	Black/African American race Medicaid/Gov't insurance/no insurance Metropolitan area > 50 miles from treatment facility	0.006 0.001 0.003 0.05
	Longer TT	Higher income	0.03

*TT, treatment time; RPA, recursive partitioning analysis; STR, sub-total resection; GTR, gross total resection; KPS, Karnofsky Performance Status; RT, radiation therapy*.

Results of bias scoring using the Newcastle-Ottawa Quality Assessment Scale for cohort studies is outlined in Table 4. Three studies received the maximum total score of 9, indicating the lowest risk of bias (27, 31, 32). The lowest score was 6/9 given to Nathan et al. indicating the highest risk of bias (29) (Table 4).

**Table 4:** This table depicts the bias score calculated for each study based on the Newcastle-Ottawa scale, with 9 as the highest score.

	**Adeberg et al. (26)**	**Blumenthal et al. (25)**	**Han et al. (27)**	**Louvel et al. (28)**	**Nathan et al. (29)**	**Noel et al. (15)**	**Osborn et al. (30)**	**Pollom et al. (31)**	**Sun et al. (32)**	**Wang et al. (33)**
Selection (Max = 4)	3	4	4	4	4	4	4	4	4	4
Comparability (Max = 2)	1	0	2	1	0	0	1	2	2	0
Outcome (Max = 3)	3	3	3	3	2	3	3	3	3	3
Total (Max = 9)	7	7	9	8	6	7	8	9	9	7

*A higher score correlates with a lower risk of bias. Factors included for comparability include Karnofsky Performance Status and extent of resection*.

## Discussion

The question of optimal timing of treatment initiation following surgical resection in patients with newly diagnosed glioblastoma has been investigated in several retrospective cohort studies, which has yielded varying results. Several studies have demonstrated decreased overall survival in these patients with increased wait time (11–13), while others have demonstrated no effect (14, 16, 34) and a third group of studies show a favorable outcome with delayed initiation of radiation therapy (17, 18). However, the majority of these studies were conducted prior to the initiation of the Stupp protocol in 2005. Many of those that were written after 2005 include subjects both before and after the Stupp era and do not provide separate analyses of Stupp patients. The addition of temozolomide to the treatment regimen for patients with glioblastoma represents an important change in the care of these patients and provided a significant survival benefit, particularly in patients with MGMT promoter methylation (2, 35). This systematic review aimed to provide an analysis of retrospective studies that only included patients receiving the current standard of care to best answer the question of optimal timing of chemoradiation therapy in glioblastoma patients in the modern era.

This study does not support an optimum time for initiation of chemoradiotherapy following surgical resection in patients with newly diagnosed HGG. The study in this systematic review with the highest number of patients evaluated (*n* = 12,738) that also received a maximum score of 9 on the risk of bias assessment found significantly improved survival in patients with a time to treatment initiation of 15–21 days in patients who underwent gross total resection (31). Five of the other publications reviewed in this study similarly found benefit to slightly longer times to treatment initiation, including the studies with the 2nd and 3rd largest sample sizes among these studies (29, 30) (*n* = 11,625 and 2,535, respectively). There are several possible explanations for the worse outcomes seen in patients with shorter TT. There is concern that starting radiation before the patient has fully recovered from surgery could result in impaired healing and an increase in radiation side effects (36–38). It is also probable that patients who start treatment sooner after surgery are chosen to do so based on the judgment of the clinician that they have more aggressive disease or worse functional status as a result of their disease. Indeed, several of the publications evaluated in this study found that patients with the shortest TT were more likely to have undergone less extensive surgery (27, 30, 33), have higher age (27, 33), have postoperative neurologic deficits, (28), have lower KPS (33), or have larger tumor size (30) compared to patients who started treatment later. All of these factors are known to have significant impact on prognosis in glioblastoma and could have contributed to the poorer survival of the early treatment group seen in several of these publications (22–24). Of the remaining four studies, three of them found no significant impact of TT on overall survival (15, 25, 28), while the 4th study, Sun et al. found that there was no survival impact with moderate TT in treatment, though significant TT > 42 days may be associated with worse outcomes (32). Although there is some regional variation, the most recent data from 2005 to 2014 showed that the majority of patients in the United States begin chemoradiotherapy within 6 weeks of surgical resection (29). With a malignancy as devastating as GBM, delays in treatment can be a concern for both patients and providers. Given the fairly narrow window in which patients are typically treated, it may be difficult to discern any significant differences in survival based on treatment timing. This systematic review provides some evidence that, in the era of the Stupp protocol, there is at least no evidence that moderate TT worsen overall patient outcomes and it is reasonable to continue the standard of treatment initiation within 6 weeks after the patient has recovered from surgery.

Each of the publications reviewed in this study suffer from the well-known limitations of retrospective studies. As ethical reasons restrict the possibility of conducting a prospective randomized trial to address this question, there are several confounding factors that have an unknown level of influence in the results of these studies. Ideally, data designed to best answer this question would include a large cohort of patients who are matched for several prognostic factors including age, extent of resection, and functional status to minimize confounders. Several of the studies included in this review attempted to simulate such a cohort by creating regression models to account for several of these prognostic factors and Pollom et al. even analyzed the data separately for patients who had biopsy only, sub-total, and gross total resections (31). Additionally, as novel treatments (such as systemic agents, immunotherapy or tumor-treating fields) are developed that could potentially improve survival of GBM patients, TT may or may not have a greater impact on OS (39). Of note, the design of several of these studies makes it difficult to evaluate the effects of significant TT in patients that may be vulnerable to treatment delays. Some of the studies had strict cutoffs and did not include patients with significant delays (11, 15, 25, 31) while others did not employ a cutoff for TT, but analyzed patients with TT >33 weeks in the same group as patients with a TT of 5 weeks, making it difficult to draw conclusions regarding this subpopulation (32). Patients who may be subject to delays in treatment, such as those who participate in inpatient rehabilitation programs after surgery and are unable to have any cancer treatment until the program is completed, may still have an impact on overall survival related to this delay and further investigation is warranted to draw a conclusion regarding this population.

This systematic review has several limitations. Due to the small number of publications that met the inclusion criteria of this study and the differing ways in which each group analyzed their data, it was not possible to create a mathematical model for evaluation of possible publication bias. The tool used to assess risk of bias for individual studies, the Newcastle-Ottawa Scale, has shown reliability between individual reviewers but has still been criticized for a paucity of evidence regarding validity of the tool (40, 41). Given the unlikelihood of a prospective trial to address this topic, a collaborative effort among institutions to review the current evidence in the Stupp protocol era is the best chance of providing an answer. Establishing a standard for grouping patients by TT and method of analysis in the future could provide a large population of studies that are directly comparable to one another.

## Author Contributions

KTW performed the initial literature search, evaluated all of the resulting articles, performed the analysis and figure generations, and wrote the manuscript. LL assisted with data representation and verification of literature search results as well as editing of the manuscript. YL assisted with editing of the final manuscript. MM assisted with literature search and editing of the manuscript. KAW generated the idea to perform this review and provided guidance to the primary author through each step of the process.

### Conflict of Interest Statement

The authors declare that the research was conducted in the absence of any commercial or financial relationships that could be construed as a potential conflict of interest.

## Supplementary Material

Table S1 This table depicts the search algorithms used for this systematic review and the number of results obtained for each database. For the EMBASE database, searches are built from the bottom up, starting with phrase #1. The final search is bolded at the top (#15) and includes all of the prior phrases.
