# Supplementary material for: The Impact of Timing of Concurrent Chemoradiation in Patients With High-Grade Glioma in the Era of the Stupp Protocol
Source: Front Oncol. 2019 Mar 27;9:186. doi: 10.3389/fonc.2019.00186 (PMC6445963; doi:10.3389/fonc.2019.00186)
Supplement: Table S1 — This table depicts the search algorithms used for this systematic review and the number of results obtained for each database. For the EMBASE database, searches are built from the bottom up, starting with phrase #1. The final search is bolded at the top (#15) and includes all of the prior phrases. [file Table_1.pdf]

Database	Date of Search	Search Algorithm	Number of Results
PubMed	07/03/2018	high grade AND glioma AND (radiotherapy OR radiation OR chemotherapy OR chemoradiotherapy OR Temozolomide) AND (time factors OR time-to-treat OR timing OR watchful waiting OR delay OR early OR optimal[tiab]) AND (craniotomy OR postoperative OR post surg* OR postoperative care)	82
EMBASE	07/03/2018	**15. #10 and #14**<br>14. #11 or #12 or #13<br>13. ‘early’<br>12. ‘delay’<br>11. ‘time’/exp OR ‘time’<br>10. #9 AND (2005:py OR 2006:py OR 2007:py OR 2008:py OR 2009:py OR 2010:py OR 2011:py OR 2012:py OR 2013:py OR 2014:py OR 2015:py OR 2016:py OR 2017:py OR 2018:py)<br>9. #1 AND #7 AND #8<br>8. #2 OR #3 OR #4<br>7. #5 OR #6<br>6. ‘postoperative period’/exp OR ‘postoperative period’ OR ‘postoperative phase’<br>5. ‘postoperative care’/exp OR ‘care, postoperative’ OR ‘postoperative care’ OR ‘postoperative therapy’ OR ‘postoperative treatment’<br>4. ‘chemoradiotherapy’/exp OR ‘chemoradiation’ OR ‘chemoradiotherapy’ OR ‘radiochemotherapy’<br>3. ‘radiation’/exp OR ‘multiple field radiation’ OR ‘non ionizing radiation’ OR ‘nonionizing radiation’ OR ‘radiation’ OR ‘radiation change’ OR ‘radiation control’ OR ‘radiation incidence’ OR ‘radiation quality’ OR ‘radiation, nonionizing’<br>2. ‘radiotherapy’/exp OR ‘bioradiant therapy’ OR ‘bucky irradiation’ OR ‘bucky radiotherapy’ OR ‘bucky ray’ OR ‘bucky ray radiation’ OR ‘bucky therapy’ OR ‘fractionated radiotherapy’ OR ‘hemibody radiation’ OR ‘hypophysectomy, radiation’ OR ‘hypophysis irradiation’ OR ‘hypophysis radiation’ OR ‘irradiation therapy’ OR ‘irradiation treatment’ OR ‘irradiation, hypophysis’ OR ‘lymphatic irradiation’ OR ‘pituitary irradiation’ OR ‘radiation beam centration’ OR ‘radiation repair’ OR ‘radiation therapy’ OR ‘radiation treatment’ ‘radio therapy OR ‘radio treatment’ OR ‘radiohypophysectomy’ OR ‘radiology, therapeutic’ OR ‘radiotherapy’ OR ‘radiotherapy setup errors’ OR ‘radiotreatment’ OR ‘roentgen irradiation, therapeutic’ OR ‘roentgen therapy’ or ‘roentgen treatment’ OR ‘rontgen therapy’ OR ‘therapeutic radiology’ OR ‘therapy, irradiation’ OR ‘therapy, radiation’ OR ‘therapy, roentgen’ OR ‘treatment, irradiation’ OR ‘treatment, radiation’ OR ‘treatment, roentgen’ OR ‘x radiotherapy’ OR ‘x ray therapy’ OR ‘x-ray therapy’<br>1. ‘glioma’/exp OR ‘brain glioma’ or ‘cerebral glioma’ OR ‘ganglioglioma’ OR ‘glia tumor’ OR ‘glia tumour’ OR ‘glial tumor’ OR ‘glial tumour’ OR ‘glioma’ OR ‘high grade glioma’ OR ‘low grade glioma’ OR ‘recurrent glioma’	493
